# Comparative mutational analysis of the Zika virus genome from different geographical locations and its effect on the efficacy of Zika virus-specific neutralizing antibodies

**DOI:** 10.3389/fmicb.2023.1098323

**Published:** 2023-02-22

**Authors:** Abdul Aziz, Muhammad Suleman, Abdullah Shah, Ata Ullah, Farooq Rashid, Sikandar Khan, Arshad Iqbal, Sisi Luo, Liji Xie, Zhixun Xie

**Affiliations:** ^1^Molecular Biology Research Center, School of Life Sciences, Central South University, Changsha, China; ^2^Centre for Biotechnology and Microbiology, University of Swat, Mingora, Pakistan; ^3^Department of Biotechnology, Shaheed Benazir Bhutto University, Upper Dir, Pakistan; ^4^New Cross Hospital, The Royal Wolverhampton NHS Trust, Wolverhampton, United Kingdom; ^5^Division of Infectious Diseases, Chongqing Public Health Medical Center, Chongqing, China; ^6^Department of Biotechnology, Guangxi Veterinary Research Institute, Nanning, China; ^7^Guangxi Key Laboratory of Veterinary Biotechnology, Nanning, China; ^8^Key Laboratory of China (Guangxi)-ASEAN Cross-Border Animal Disease Prevention and Control, Ministry of Agriculture and Rural Affairs of China, Nanning, China

**Keywords:** mutational analysis, neutralizing antibodies, Guillain–Barré syndrome, MD simulations, Zika virus

## Abstract

The Zika virus (ZIKV), which originated in Africa, has become a significant global health threat. It is an RNA virus that continues to mutate and accumulate multiple mutations in its genome. These genetic changes can impact the virus’s ability to infect, cause disease, spread, evade the immune system, and drug resistance. In this study genome-wide analysis of 175 ZIKV isolates deposited at the National Center for Biotechnology Information (NCBI), was carried out. The comprehensive mutational analysis of these isolates was carried out by DNASTAR and Clustal W software, which revealed 257 different substitutions at the proteome level in different proteins when compared to the reference sequence (KX369547.1). The substitutions were capsid (17/257), preM (17/257), envelope (44/257), NS1 (34/257), NS2A (30/257), NS2B (11/257), NS3 (37/257), NS4A (6/257), 2K (1/257), NS4B (15/257), and NS5 (56/257). Based on the coexisting mutational analysis, the MN025403.1 isolate from Guinea was identified as having 111 substitutions in proteins and 6 deletions. The effect of coexisting/reoccurring mutations on the structural stability of each protein was also determined by I-mutant and MUpro online servers. Furthermore, molecular docking and simulation results showed that the coexisting mutations (I317V and E393D) in Domain III (DIII) of the envelope protein enhanced the bonding network with ZIKV-specific neutralizing antibodies. This study, therefore, highlighted the rapid accumulation of different substitutions in various ZIKV proteins circulating in different geographical regions of the world. Surveillance of such mutations in the respective proteins will be helpful in the development of effective ZIKV vaccines and neutralizing antibody engineering.

## 1. Introduction

Zika virus (ZIKV), a mosquito-borne flavivirus, was first isolated in 1947 from a serum sample of rhesus monkeys during an investigation of yellow fever virus (YFV) in the Zika forest of Uganda (Dick et al., 1952). Other human pathogenic flaviviruses include dengue virus (DENV), West Nile virus (WNV), and Japanese encephalitis viruses (JEV) (Kuno et al., 1998). Zika infections usually cause asymptomatic and mild febrile infections associated with vomiting, fever, rash, sweating, pain in the muscles and the back of the eyes, and conjunctivitis, but severe neurological phenotypes such as Guillain–Barré syndrome and congenital Zika syndrome have also been reported (Carteaux et al., 2016; Rubin et al., 2016). In infected pregnant women and mice, ZIKV can be transmitted to the fetus, leading to severe fetal abnormalities such as microcephaly, spontaneous abortion, and intrauterine growth restriction (Brasil et al., 2016; Cugola et al., 2016; Miner et al., 2016). Sexual and blood-born transmission of ZIKV has also been reported, making the virus a major threat to public health worldwide (Musso et al., 2015; Gregory et al., 2017).

The genome of ZIKV consists of positive-sense, single-stranded RNA with 10,794 bases, forming a long open reading frame (ORF) flanked by a 5′UTR and 3′ UTR. The RNA genome is 5′ capped but lacks a 3′ poly-A tail. The viral RNA is translated into a large precursor of 3,423 amino acid polyprotein that is co and post-translationally cleaved to yield three structural proteins [the capsid (C), precursor membrane (preM), and envelope protein (E)] and eight non-structural (NS) proteins (NS1, NS2A, NS2B, NS3, NS4A, 2K, NS4B, and NS5) (Mukhopadhyay et al., 2005; Ye et al., 2016). The 5′ terminus has a methylated nucleotide cap, and the 3′ terminus has a loop structure that plays a role in cellular translation. Structural proteins are involved in viral particle assembly, while non-structural proteins are responsible for viral replication, assembly, and evasion from the host immune system.

When a virus adapts to a new host, it exploits the host’s cellular machinery for its successful entry, replication, and evasion of the host’s immune responses (Rampersad and Paula, 2018). To achieve these goals, the virus must undergo continuous mutations in its genome to modify antigenic epitopes on its proteins. RNA viruses, including ZIKV, have characteristic features of high mutability in their genomes, which in turn increases the virulence and transmission of the viruses (Rashid et al., 2022). Mutations in the structural protein of ZIKV have been reported to be involved in various biological pathways. For instance, a mutation in the preM protein (S139N) has been shown to increase neurovirulence in neonatal mice (Yuan et al., 2017). Similarly, three mutations in the E protein of ZIKV, D683E, V763 M, and T777 M, affect the stability of the E protein, binding the virus particle to its receptor, and are associated with a higher incidence of congenital Zika syndrome (Pettersson et al., 2016; Weaver, 2017). Interestingly, DIII (AA299-403) of the E protein of ZIKV is reported to be targeted by many neutralizing antibodies (Zhao et al., 2016). Likewise, two mutations (A982 V and T233A) have been reported in the NS1 protein of ZIKV. The substitution A982 V in NSI not only increased the secretion of NS1 in the circulatory system and enhanced transmission of ZIKV from mice to mosquitos but also reduced the phosphorylation of TBK1 and led to the inhibition of interferon-beta (ß-IFN) and facilitated viral replication (Liu et al., 2016; Xia et al., 2018). Similarly, the substitution T233A in NS1 isolated from the human microcephalic fetus has been reported to destabilize the conformation of NSI and may affect viral replication and pathogenesis (Weaver, 2017). Similarly, a single mutation (A2283S) in NS4B and three mutations (A/T3046I, G/R3107 L, and R/S3167N) in the RNA-dependent RNA polymerase (NS5) may inhibit the interferon regulatory pathway and increase viral replication (Pettersson et al., 2016). As an RNA virus, ZIKV might have a rapid rate of mutation, and the accumulation of coexisting mutations in multiple or single genes might alter its virulence, infectivity, or transmissibility and may also lead to immune evasion and drug resistance. Hence, establishing the coexisting mutation atlas of the ZIKV genome can provide valuable information for assessing the mechanisms linked to pathogenesis, immune modulation, and viral drug resistance.

Since the genomes of ZIKV isolates have been sequenced by many countries and submitted to the National Center for Biotechnology Information (NCBI) database, in this study, we attempted to investigate and identify insertions, deletions and substitutions at the proteome level in the genomes of ZIKV from different geographical regions of the world. We also elaborated the effects of these mutations on the efficacy of already-reported ZIKV-specific neutralizing antibodies using molecular docking and simulation approaches.

## 2. Materials and methods

### 2.1. Retrieval of the ZIKV genome sequences

The amino acid sequences of the structural and non-structural proteins of ZIKV deposited in the NCBI^1^ repository were downloaded. A total of 175 complete genome sequences of ZIKV have been deposited in the NCBI by 35 countries and territories. Only the coding and complete gene sequences were used in the present study.

### 2.2. Multiple sequence alignment

Multiple sequence alignment of the three structural (capsid, pre-M and E) and eight non-structural proteins (NS1, NS2A, NS2B, NS3, NS4A, 2K, NS4B, and NS5) of 175 ZIKV isolates was conducted using Lasergene software package (DNASTAR, Madison, WI) (7.1) and Clustal W program of the MEGA software (7.1) concerning already reported prototype KX369547.1 of ZIKV.

### 2.3. Sequence-based structural stability prediction

The I-mutant^2^ and MUpro^3^ online sequence-based tools were used to analyze the structural stability change of the top reoccurring mutations in both structural and non-structural proteins of ZIKV by keeping all the parameters in default.

### 2.4. Wild structure retrieval and variant modeling

The wild-type structure of the E protein of ZIKV (5JHM) was retrieved from the protein data bank. For the construction of the mutant version of the E protein of ZIKV, Chimera 1.15 software was utilized.

### 2.5. Docking of wild-type and mutant E protein with neutralizing antibodies

The High Ambiguity Driven Protein–protein Docking (HDOCK)^4^ online server algorithm was used for interaction of both wild-type and a mutant version of the E protein of ZIKV and previously reported neutralizing antibodies ZV-64 (PDB, 5KVF) and ZV-67 (PDB, 5KVG) to investigate the bonding efficiencies (Zhao et al., 2016).

### 2.6. Molecular dynamics simulation of the E protein and neutralizing antibody complexes

Molecular dynamic simulation (MD) of wild-type E protein with two complexes of neutralizing antibodies (E Wild-type ZV-64, E Wild-type ZV-67) and a mutant version of the E protein with the same neutralizing antibody (E-Mutant-type ZV-64, E-Mutant-type ZV-67) complexes was completed by using the AMBER20 simulation package (Case et al., 2005; Roe and Cheatham, 2013). The default parameters used by Suleman et al. were employed to complete the 50 nano second (ns) simulation for each complex. Post-simulation analyses, such as Root Mean Square Deviation (RMSD) and Root Mean Square Fluctuation (RMSF), were calculated using CPPTRAJ and PTRAJ modules (Suleman et al., 2022).

## 3. Results

### 3.1. Worldwide sequenced genomes of ZIKV deposited in NCBI

**FIGURE 1:**
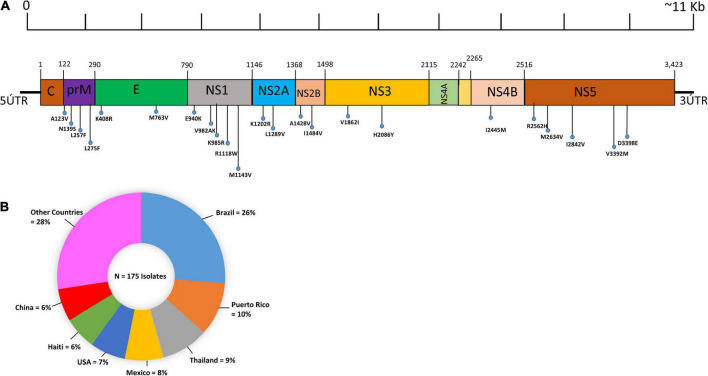
Pictorial representation and geographic distribution of ZIKV genome sequences. **(A)** Pictorial representation illustrating the distribution of recurrent mutations and hypervariable genomic hot spot mutations in structural (C, preM, E) and non-structural proteins (NS1, NS2A, NS2B, NS3, NS4A, NS4B, and NS5) along with the ZIKV genome. The genomic hotspots are represented by vertical lines. **(B)** Geographic distribution of the 175 complete genome sequences of ZIKV. The pie chart represents the percentage of genomes of the ZIKV genomes sequenced according to their geographic origins. The colors indicate different countries.

In the current study, we have attempted to show the geographic origins of these submitted ZIKV genomes, which were fully sequenced. Only coding regions of the ZIKV genome were examined in this study for mutational analysis at the proteome level. The genomic organization along with the polyprotein (structural and non-structural proteins) of ZIKV is shown in Figure 1A. The results of the present study showed that the most abundantly complete genome sequence of the ZIKV originated from Brazil (24%), followed by Thailand (8%), while only one ZIKV genome has been sequenced by Argentina (Figure 1B).

### 3.2. Identification of the top ten mutated isolates of ZIKV

**FIGURE 2:**
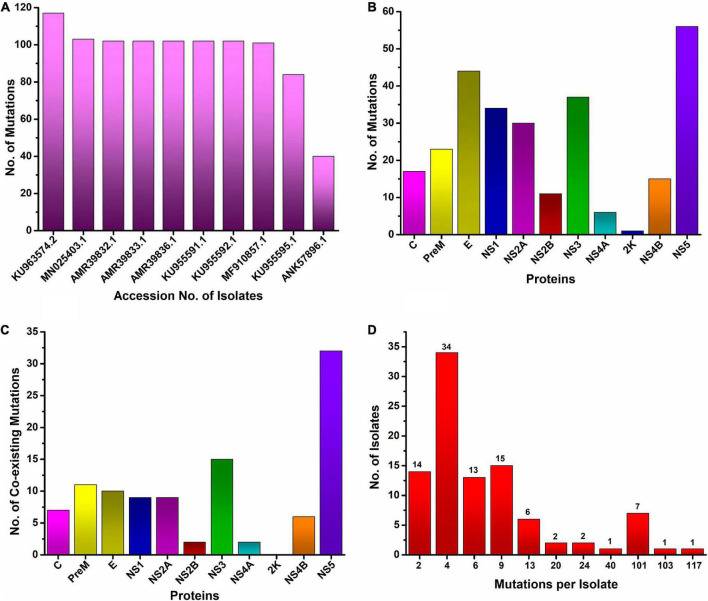
Worldwide mutational analysis of the ZIKV genome per isolate **(A)**. Top 10 most mutated ZIKV isolates along with accession no. **(B)**. Frequency distribution of substitution in each protein of ZIKV **(C)**. Number of coexisting mutations in each protein of the ZIKV. **(D)** Frequency distribution of ZIKV isolates harboring varying numbers of coexisting mutations.

In this study, the top ten mutated ZIKV isolates at the protein level were identified. We found that the most mutated isolate was KU963574.2 (Nigeria), harboring 111 substitutions along with six deletions (T446-G447-H448-E449-T450-D451), followed by MN025403.1 (Guinea), which had a total of 103 different substitutions. Similarly, seven isolates were identified from Senegal, where five isolates (AMR39832.1, AMR39833.1, AMR39836.1, KU955591.1, and KU955592.1) harbored 102 substitutions, while two isolates, MF510857.1 and KU955595.1, had 101 and 84 mutations, respectively. Likewise, one isolate from Malaysia ANK57896.1 carried 40 different substitutions in different proteins (Figure 2A).

### 3.3. Identification and analysis of mutations in structural proteins of ZIKV isolates circulating in different geographical regions of the world

The three structural proteins of ZIKV, capsid C, preM, and envelope (E), are not only involved in the assembly of mature virions and successful ingress and egress of the host cells, but the latter two (PreM and E) are being used as antibody-activating epitopes in ZIKV vaccine development (Robbiani et al., 2017). To identify the mutational profile at the protein level (accumulated through natural selection) in structural proteins of ZIKV, multiple sequence alignments of 175 isolates of ZIKV were carried out from 35 countries and territories that had been deposited in the NCBI repository. We found mutations in all three structural proteins, i.e., Capsid (C), preM, and envelop (E). Collectively, 67 different mutations were observed in these three proteins when compared to the reference strain sequence KX369547.1 (Supplementary Table 1).

#### 3.3.1. Mutational analysis of the capsid (C) protein

The C protein is 122 amino acids (AAs) in length with a molecular weight of 11 kDa. Multiple copies of the capsid protein encapsulate the genomic RNA of ZIKV and form nucleocapsid core particles of mature ZIKV (Panwar and Singh, 2018). The complete CoDing Sequence (CDS) of the capsid protein appears in all 175 isolates of ZIKV in the NCBI database. The present study revealed that among 175 isolates of ZIKV, 67 isolates harbored 17 different amino acid mutations in the capsid protein, with a signature A106T (35%) mutation, which is indicative of widespread mutation in the C protein of ZIKV circulating worldwide in comparison to the reference isolate sequence KX369547.1 (Figure 2B, Table 1 and Supplementary Table 1). This mutation was predominant (83%) in Cambodian isolates of the ZIKV. The other less common mutations that coexist with A106T in the C protein of ZIKV were K6E, D7E, S8I, G9R, G10R, and F111I in the EU545988.1 (Micronesia) isolate. The second most mutated C protein of the ZIKV isolate was found at KU963574.2 (Nigeria), having seven coexisting mutations, i.e., S25N, F27L, K101R, T108A, V110I, G104S, and A120V when compared to the reference isolate sequence KX369547.1 (Figure 2C). Likewise, the most reoccurring mutations observed in the C protein were A106T and D107E, while no insertions or deletions were identified in the C protein (Table 1). D107E in the capsid protein was previously reported mostly in Colombia, Panama, and Venezuela (Metsky et al., 2017).

**TABLE 1:** Summary of mutations in each protein of the ZIKV.

Protein	No. of amino acids	No. of available isolates	No. of mutated isolates	No. of mutations	Rate of mutation	Indel mutation	Top reoccurring mutations
Capsid	122	175	67	17	0.10	–	A106T, D107E
PreM	168	175	27	23	0.13	–	A123V, L151M, K242R, L257F, L25F, L275F
Envelop	500	175	110	44	0.25	10 (del)	V313I, K498R, M763V, M777T
NS1	352	175	146	34	0.20	–	Y916H, E920L, V982A, K985R, M1143V R1118W, M1143V
NS2A	226	175	24	30	0.17	–	K1202R, L1274P, L1289V
NS2B	130	175	31	11	0.06	–	A1428V, T1477A, I1484V
NS3	617	175	108	37	0.21	–	H1857Y, V1862I, H1902N, M207L, H2086Y.
NS4A	127	175	76	06	0.10	–	F2123L
2K	23	175	01	01	0.006	–	–
NS4B	251	175	14	15	0.10	–	I2295M, I2376M, I2445M
NS5	903	175	136	56	0.32	–	R2562H, M2634V, I2842V, R3045C, S3162P, T3353A, V3392M, D3398E, M3403V

#### 3.3.2. Mutational analysis of the preM protein

The structural preM protein of the ZIKV is 168 AAs in length with 8 kDa. Initially, after translation, this protein is in an immature form, but later, a cellular furin-like protease cleaves it into prepeptide and M protein. The M protein forms a complex with the envelope protein and protects the E protein (Panwar and Singh, 2018). The M protein has been used for vaccine development against ZIKV infection (Reichmuth et al., 2016). The present results showed that among 175 isolates of ZIKV, 27 isolates had 23 different substitutions in the preM protein (Figure 2B). Among these mutations, the L257F substitution was the most reoccurring mutation in the preM protein. Similarly, other less reoccurring mutations identified in the preM protein were A123V, S130N, N139S, E143K, L151M, K242R, L257F, and L275F (Table 1). Likewise, 11 coexisting mutations (V125I, N139S, E143K, P148A, M153V, Y157H, I158V, R246K, L257F, A260V, and A262V) were exhibited by two isolates, MN025403.1 (Guinea) and KU963574.2 (Nigeria) (Figure 2C and Supplementary Table 1). In addition, no deletion or insertion was observed in the preM protein of ZIKV with respect to the reference sequence KX369547.1 (Table 1).

#### 3.3.3. Mutational analysis of the envelope (E) protein

Among the structural proteins of ZIKV, the E protein is the main and largest surface glycoprotein of virions, having 500 AAs with a molecular weight of 53 kDa (Panwar and Singh, 2018). The E protein of ZIKV is one of the major proteins involved in receptor binding and fusion of the virus with host cells. A compact ZIKV particle is composed of 180 copies of the E protein, and each E protein of ZIKV is composed of three domains, namely Domain I (DI), which contains the N-terminus of the E protein; Domain II (DII), which is involved in dimerization of the E protein; and DIII, which mediates attachment to target cells. The E protein is used for vaccine development, and many ZIKV neutralizing antibodies bind to Domain III (303AA-407AA) of the E protein, thus suggesting that Domain III is an important target for neutralizing antibodies (Robbiani et al., 2017). This study reports that among the structural proteins of ZIKV, the E protein was observed to be the most susceptible to mutations, followed by preM and C proteins harboring a total of 44, 23, and 17 different mutations, respectively (Figure 2B and Table 1). The most reoccurring mutation in the E protein is M763V, followed by V313I, K408R, and M777T (Table 1). Furthermore, two segments comprising four AAs and six AAs, N444-D445-T446-G447 and T446-G447-H448-E449-T450-D451, were deleted in isolates KY553111.1 (Republic of Korea) and KU963574.2 (Nigeria), respectively; however, no insertion was observed. Similarly, 10 coexisting mutations (A410T, I459V, F575S, I607V, E683D, A727V, L728F, M763V, M777T, and L785M) were observed in isolate MN025403.1 (Guinea) (Figure 2C and Supplementary Table 1).

### 3.4. Identification and analysis of mutations in non-structural proteins of ZIKV strains circulating in different geographical regions of the world

The ZIKV genome encodes eight non-structural proteins, including NS1, NS2A, NS2B, NS3, S4A, 2K, NS4B, and NS5. These non-structural proteins are involved in the viral replication complex inside the infected host cell. In the present study, a total of 190 substitutions were identified in the non-structural proteins of 175 isolates of ZIKV genomes deposited in the NCBI repository from 35 different countries throughout the world. The following are the details of mutations in non-structural proteins of ZIKV.

#### 3.4.1. Non-structural protein 1 (NS1)

The NS1 protein of the ZIKV has 351 AAs with a molecular weight of approximately 48 kDa (Panwar and Singh, 2018). It is a secretory protein involved in genome replication as well as in host immune evasion by ZIKV through modulating the host immune mechanism (Mackenzie et al., 1996; Brown et al., 2016). In the present study, 34 different AAs substitutions at different positions in NSI were identified in 146 isolates (Figure 2B, Table 1 and Supplementary Table 1). Among these substitutions, nine coexisting mutations (D846E, R863K, S886P, E940K, V956I, V988A, K1007R, I1030V, and M1058V) were observed in isolate MN025403.1 (Guinea) (Figure 2C). In NS1, the most recurring mutation was E940L in 44 isolates followed by M1143V in 17 isolates. Other reoccurring mutations include Y916H, V982A, K985R, M1143V, R1118W, and M1143V (Table 1). Previously, it has been reported that substitution at V982A reduced secretion of NS1 to the host circulatory system, thus leading to reduced uptake by the mosquito and increased interferon inhibition by NS1 (Liu et al., 2016). Similarly, R1118 and M1143V were first reported in Brazilian isolates (Faria et al., 2017). However, the present study identified these two mutations (R1118 and M1143V) in isolates from Haiti, Martinique, and Panama. Furthermore, no insertion or deletion was observed in the NS1 protein (Table 1 and Supplementary Table 1).

#### 3.4.2. Non-structural protein (NS2A)

The NS2A protein of the ZIKV has 226 AAs with a molecular weight of 22 kDa. NS2A induces endoplasmic reticulum (ER) membrane rearrangement, interacts with NS3 and NS5 of ZIKV and mediates replication and capsid assembly of ZIKV particles (Panwar and Singh, 2018). Among 175 isolates of ZIKV, 24 isolates had 30 different substitutions at different positions (Figure 2B and Table 1), while nine coexisting mutations (I1180M, I1191V, A1204V, I1226V, D1270E, I1275V, V1289A, T1297A, and L1354M) (Figure 2C and Supplementary Table 1) were identified in isolate MN025403.1 (Guinea). Similarly, two different reoccurring and coexisting mutations, K1202R and L1289V were identified in 15 Brazilian isolates of ZIKV (Supplementary Table 1). The most frequently recurring mutations in NS2A were K1202R and L1289V followed by L1274P (Table 1).

#### 3.4.3. Non-structural protein (NS2B)

The NS2B protein of ZIKV is 130 AAs with a molecular weight of approximately 14 kDa that have been reported to interact with the NS3 protein and be involved in viral replication (Panwar and Singh, 2018). The present study revealed that among 175 isolates of ZIKV, only 31 isolates had 11 different substitutions of AAs in the NS2B protein (Figure 2B and Table 1). Among these substitutions, A1428V was observed in 15 Brazilian isolates (Supplementary material). Interestingly, the A1428V substitution in NS2B of these isolates coexisted with K1202R and L1298V in the NS2A protein of these isolates (Supplementary Table 1). The other reoccurring mutations in different isolates were S1417I, D1461E, and T1477A (Table 1). Only two coexisting mutations, D1461E and T1477A, were observed in isolate MN025403.1 (Guinea) (Figure 2C), while no insertion or deletion was observed in NS2B during this study (Table 1).

#### 3.4.4. Non-structural protein (NS3)

The NS3 protein of the ZIKV is 617 AAs, having a molecular weight of approximately 70 kDa. which has been reported to play many important roles in viral RNA replication and methylation processes (Panwar and Singh, 2018). This study showed that among 175 isolates of ZIKV, 67 isolates had 37 different substitutions of AAs in the NS3 protein (Figure 2B and Table 1). Among these substitutions, V1862I was observed in 25 isolates, followed by H2086Y in 16 isolates. This V1862I substitution in NS3 coexisted with A1428V of NS2A and K1202R and L1298V of NS2B in 15 Brazilian isolates (Supplementary Table 1). Similarly, 15 coexisting mutations (S1558A, H1594L, I1658V, R1671V, K1687R, T1717K, V1722A, T1753I, K1860R, V1862I, H1902N, V1909I, L1974M, R2085K, and H2086Y) were observed in isolate MN025403.1 (Guinea) (Figure 2C and Supplementary Table 1). In addition, the five most reoccurring mutations were identified, while no deletion or insertion was observed in the NS3 protein during this study (Table 1).

#### 3.4.5. Non-structural protein (NS4A)

The NS4A protein of the ZIKV is 127 AAs long, having a molecular weight of approximately 16 kDa, which has been reported to play a key role in the localization of the replication complex toward the membrane and the processing of polyproteins of the ZIKV inside the host cell (Panwar and Singh, 2018). The present study showed that among 175 isolates of ZIKV, 17 isolates had 6 different substitutions of amino acids in the NS4A protein (Figure 1B and Supplementary Table 1). Among these substitutions, 2 coexisting mutations, F2123L, and F2127D, were observed in isolate MN025403.1 (Guinea), while no deletion or insertion mutation was observed in the NS4A protein. Similarly, only one mutation, V2259I, was observed in the 2K protein in isolate EU545988.1, while no insertion or deletion was observed in the NS2A and 2K proteins in the present study (Figure 2C and Table 1).

#### 3.4.6. Non-structural protein (NS4B)

The NS4B protein of ZIKV is a small hydrophobic membrane-associated protein of 251AA with a size of 27 kDa. The NS4B protein along with the NS3 protein plays a significant role in ZIKV genome replication (Panwar and Singh, 2018). The present study showed that out of 175 isolates of ZIKV, only 14 isolates have 15 different substitutions of amino acids in the NS4B protein. Among these substitutions, 6 coexisting mutations (L2282, R2289K, A2293T, F2318L, I2453V, S2455L) were observed in isolate MN025403.1 (Guinea). Moreover, the most reoccurring mutations were I2295M, I2367M, and I2445M, while no deletion or insertion mutation was observed in the NS4B protein during the entire study (Figure 2C, Table 1 and Supplementary Table 1).

#### 3.4.7. Non-structural protein (NS5)

NS5 is the largest protein among all non-structural proteins of ZIKV. It has 903 AAs 103 kDa in size. This has a dual role; it acts as a methyl and guanylyltransferase (MTase and GTase) as well as an RNA-dependent RNA polymerase; thus, it is an important target for drug development against ZIKV infection (Panwar and Singh, 2018). In the present study, out of 175 isolates, 136 had 56 different substitutions of AAs in NS5. Among these mutations, 32 coexisting mutations (Y2594H, I2598V, K2621R, A2679T, L2715M, Y2722H, T2749I, V2787A, N2800R, S2807N, I2842V, S2896N, H2909R, E2935V, Q2969H, V3039I, S3034N, I3046A, R3065K, K3080E, I3089V, K3107G, Q3154H, R3161K, S3162P, H3167R, N3172D, D3223S, S3304A, V3333M, T3353K, and N3387D) were observed in isolate MN025403.1 (Guinea) (Figure 2C). Similarly, reoccurring mutations were abundant in NS5, while no deletion or insertion was examined during this study when compared to the reference sequence (Table 1 and Supplementary Table 1).

### 3.5. Identification of highly mutated proteins of ZIKV

The ZIKV genome encodes three structural and seven non-structural proteins. Among the three structural proteins in all 175 isolates of ZIKV, the E protein was the most mutated protein, having a total of 44 different substitutions, followed by the pre-M protein, having 23 different substitutions. Similarly, among the non-structural proteins, NS5 is the most mutated protein, having 56 different substitutions, followed by the NS3 protein, which harbors 37 different substitutions at various positions. The 2K non-structural protein was found to be the least mutated protein, having only one substitution in only one isolate, EU545988.1 (Micronesia) (Figure 2B and Table 1).

### 3.6. Identification of hypervariable genomic hotspots

Interestingly, among the reoccurring mutations in three structural proteins, pre-M protein has three A123V, N139S, L257F, E protein has two K408R, M763V while no hypervariable hotspot was observed in the C protein. Similarly, among the non-structural proteins, NSP1 (E940K, V982A, K985R, R1118W, M1143V) and NSP5 (R2562H, M2634V, I2842V, V3392M, D3398E) have five hotspots, NS2A (K1202R, L1289V), NS2B (A1428V, I1484V), and NS3 (V1862I, H2086Y) each have two hotspots, NS4B has one (I2445M), and NS4A and K have no hotspots (Figure 1B).

### 3.7. Occurrence of coexisting mutations in ZIKV isolates

Analysis of mutational events per isolate of the ZIKV genome revealed a maximum of 111 coexisting substitutions and six (T446-G447-H448-E449-T450-D451) deletions in one isolate KU963574.2 from Nigeria followed by 103 coexisting mutations in the MN025403.1 (Guinea) isolate. Similarly, a maximum of 102 coexisting mutations were observed in five isolates from Senegal (AMR39832.1, AMR39833.1, AMR39836.1, KU955591.1, KU955592.1), while the maximum number of isolates had four mutations in their genome (Figure 2D).

### 3.8. Effects of the most frequent reoccurring mutations on the stability of structural and non-structural proteins of the ZIKV isolates

The I-mutant and MUpro online servers revealed only one substitution, H1857Y, in the NS3 protein among non-structural proteins and showed increased stability, while the rest of the substitutions in both structural and non-structural proteins showed decreased stability of their respective proteins (Supplementary Table 2).

### 3.9. Mutation modeling and RMSD calculation of the envelope protein (E) of ZIKV

**FIGURE 3:**
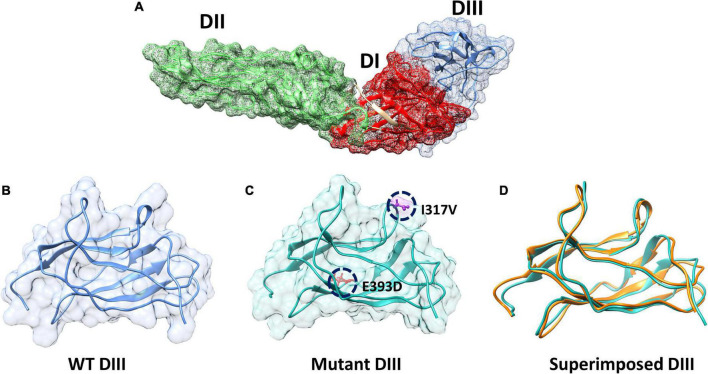
Domains architecture of ZIKV E protein and mutant modeling. **(A)** Domain mapping of ZIKV E protein. **(B)** Wild-type DIII of the E protein. **(C)** Mutant (I317V, E393D) DIII. **(D)** Superimposition of wild-type DIII on mutant DIII to calculate the RMSD.

The envelope protein (E), of ZIKV is one of the major proteins involved in receptor binding and fusion of the virus with the host cell, and it also elicits type-specific neutralizing antibodies (Wu et al., 2017). The E protein of ZIKV is composed of three domains: Domain I (DI), which contains the N-terminus of the E protein; Domain II (DII), which is involved in dimerization of the E protein; and DIII, which mediates attachment to target cells (Robbiani et al., 2017). These three domains in the E protein of ZIKV are arranged in such a way that DI is located in the center, while DII and DII flank the two sides of the DI and form a monomer (Figure 3A). Furthermore, each monomer of the E protein interacts with the adjacent monomer of the E protein in an antiparallel manner and forms a dimer (Figure 3B) (Kostyuchenko et al., 2016). DIII of the E protein is reported to be the main target for neutralizing antibodies. To determine the mechanism of hydrogen bonding strength between the wild-type and mutant ZIKV DIII, which are responsible for binding with neutralizing antibodies, we used chimera software to model the shortlisted mutations (I317V, E393D) in the DIII of the wild-type structure (Figure 3C). Afterward, we superimposed the wild-type and mutant DIII to visualize the effect of the generated mutations on the structural confirmation and recorded the RMSD values. The RMSD value for the superimposed structure was 0.8 Å, which showed the perturbation of wild-type structural elements, structural deviations, and changes in the protein conformation (Figure 3D).

### 3.10. Bonding network analysis using molecular docking of E protein with neutralizing antibodies

**FIGURE 4:**
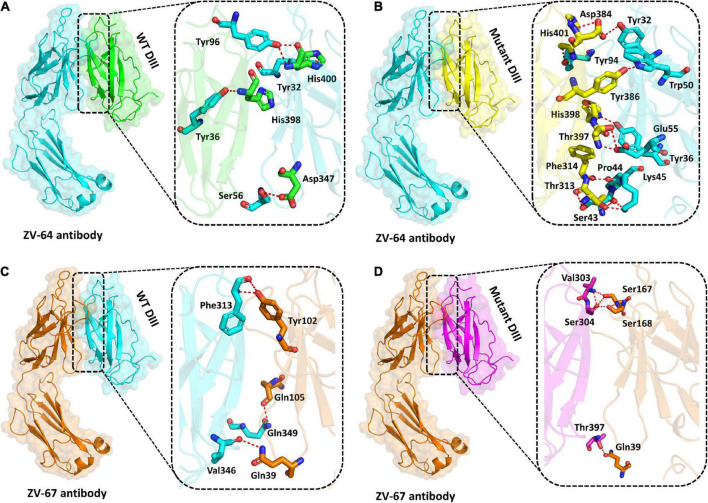
Docking of wild-type and mutant DIII with ZV-64 and ZV67 neutralizing antibodies. **(A)** Docking of wild-type DIII with ZV-64 neutralizing antibody. **(B)** Docking of mutant DIII with the ZV-64 neutralizing antibody. **(C)** Docking of wild-type DIII with the ZV-67 neutralizing antibody (DIII). **(D)** Docking of mutant DIII with ZV-67 neutralizing antibody.

Previously, it has been reported that many ZIKV antibodies bind to Domain III (303AA-407AA) of the E protein of ZIKV, thus suggesting that Domain III is an important target for neutralizing antibodies (Robbiani et al., 2017). Structural binding of the wild-type and mutant DIII with neutralizing antibodies ZV-64 and ZV-67 was performed to visualize the effect of mutation on the binding efficacy with the antibodies (Figure 4). Docking of the wild type and mutant by the HDOCK online server revealed key differences in the binding affinity and contact network. The interaction interface analysis of the wild-type DIII-ZV-64 complex predicted by HDOCK through PDBsum revealed that there are five hydrogen bonds formed between the DIII-ZV-64 complexes. The AAs residues that formed hydrogen bonds in the DIII-ZV-64 complex are His400-Tyr96 (double hydrogen bonds), His400-Tyr32, His398-Tyr36, and Asp347-Ser56 (Figure 4A). However, the analysis of the interaction interface of the mutant DIII-ZV-64 complex revealed that 11 hydrogen bonds exist among the aforementioned complexes. The residues involved in hydrogen bond formation are His400-Tyr94, His398-Tyr36, Tyr386-Trp50, Asp384-Tyr32, Thr397-Glu55, Phe314-Pro44, Thr313-Lys45, and Thr313-Ser43 (Figure 4B). Hence, the data revealed that mutations in DIII enhanced the binding of ZIKV E protein with the neutralizing antibody ZV-64. To further confirm the above data, we also checked the effect of the identified mutations in the DIII of the ZIKV E protein on binding with another neutralizing antibody called ZV-64. After docking the wild-type DIII with the ZV-67 antibody, the analysis of the interaction interface identified 3 hydrogen bonds between wild-type DIII and the ZV-67 antibody. The residues involved in hydrogen bond formation are Gln349-Trp103, Val346-Gln39, and Phe313-Tyr102 (Figure 4C). However, the interaction interface of the mutant DIII-ZV-67 complex revealed one salt bridge and four hydrogen bonds. The hydrogen bonds formed between Thr397-Gln39, Val303-Ser168, Ser304-Ser168, and Ser304-Ser167 residues while a salt bridge formed between Lys394-Glu150 residues. These data confirmed that the mutations in the domain (DIII) enhanced the binding of ZIKV E protein with the neutralizing antibodies, suggesting that the mutations in DIII boosted the hydrogen bonding between the epitopes in DIII and neutralizing antibodies ZV-64 and ZV-67. Nevertheless, strong hydrogen bonding between an antigen and antibody is a prerequisite for neutralization of the antigen.

### 3.11. Analysis of dynamics stability and flexibility

**FIGURE 5:**
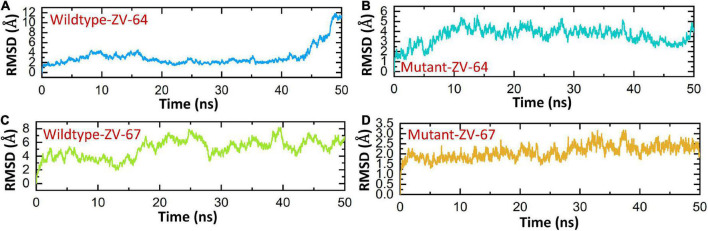
Calculation of RMSD for all complexes. **(A)** Wild-type DIII-ZV-64 complex. **(B)** mutant DIII-ZV-64 complex, **(C)** wild-type DIII-ZV-67 complex, and **(D)** mutant DIII-ZV-67 complex.

A 50 ns simulation was performed to check the dynamic behavior of both wild-type ZV and mutant ZV antibody complexes. Figures 5A–D shows that the mutant complexes are more stable than the wild type. Figure 5A shows that the wild-type ZV-64 complex gains stability at 9 ns and remains stable until 40 ns, but after this, no major perturbation was recorded in the RMSD value until 50 ns. On the other hand, Figure 5B shows that the mutant-ZV-64 complex attained stability at 10 ns and remained stable until 50 ns with an average RMSD of 3 Å. Similarly, the wild-type ZV-67 complex gained stability at 2 ns and remained stable until 15 ns; however, a sudden rise in RMSD was depicted at 15 ns and then gained stability at 30 ns until 50 ns with an average RMSD of 6 Å. In the case of the mutant-ZV-67 complex, the system equilibrated at 3 ns with an average RMSD of 2.5 Å; however, after the system equilibration, no significant fluctuations were seen during the simulation time frame of 50 ns. A lower RMSD represents higher complex stability, whereas a higher RMSD shows lower stability. In summary, the simulation results support the conclusion that the mutant complexes are more stable than the wild type, as indicated by the lack of significant fluctuations in the RMSD values. The RMSD results also suggest that the mutations in the ZIKV enhances the binding affinity with the neutralizing antibodies. Overall, these results provide further evidence that the mutations in ZIKV improves its binding to the neutralizing antibodies, and suggest that the mutant ZIKV may be a more appropriate target for the development of therapeutics.

**FIGURE 6:**
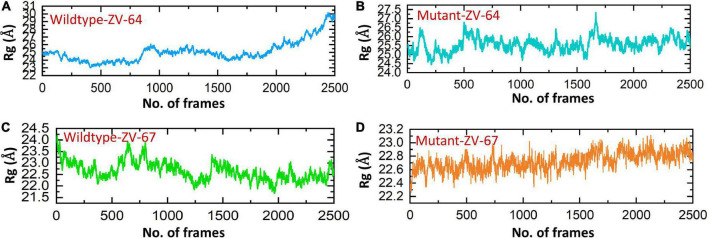
Calculation of Rg for all complexes. **(A)** Rg value for the wild-type DIII-ZV-64 complex, **(B)** Rg value for the mutant DIII-ZV-64 complex, **(C)** Rg value for the wild-type DIII-ZV-67 complex, and **(D)** Rg value for the mutant DIII-ZV-67 complex.

Afterward, the radius of gyration (Rg) was calculated to check the compactness of the aforementioned complexes (Figures 6A–D). A higher Rg value indicates that the complex is unstable, while a lower Rg value represents the stability of the complexes. As shown in Figure 6, the average Rg values for wild-type ZV-64 and mutant ZV-64 were 27 and 25, respectively, while the average Rg values for wild-type ZV-67 and mutant ZV-67 were 23 and 22, respectively. These results also verified that the mutant complexes have lower residual fluctuations in the interacting residues. The values of Rg for the four complexes are shown in Figure 6.

**FIGURE 7:**
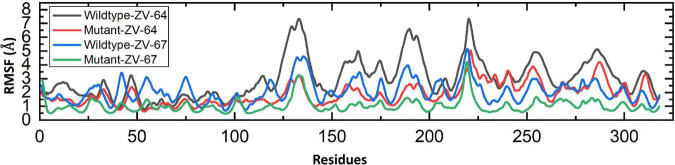
RMFS calculated for the residual flexibility of all four complexes.

Finally, the root-mean-square fluctuation (RMSF) was calculated to check the fluctuation at the residual level. The lower RMSF value shows high stability, while the higher value represents the instability of complexes. As shown in Figure 7, the average RMSF values of mutant complexes are lower than those of the wild-type complexes, which further confirmed the results of RMSD and Rg. The RMSF values for all complexes are shown in Figure 7.

## 4. Discussion

ZIKV was first isolated from Uganda in 1947 and eventually spread to several countries and territories of the world. Similar to other RNA viruses, ZIKV has undergone several mutations and accumulated various mutations in structural and non-structural proteins. These mutations have enabled it to adapt to various epidemiological settings. In this study, a comprehensive genome-wide mutational analysis of 175 ZIKV isolates, available in the NCBI database, deposited by 35 different countries and territories of the world was carried out. This study reports all the reoccurring and coexisting mutations in both structural and non-structural proteins of ZIKV isolates. In addition, the effect of mutations (specifically mutations in domain III of the E protein) on the efficacy of already reported neutralizing antibodies was also evaluated.

Among the structural proteins of ZIKV, the preM and envelope (E) proteins have been used for the development of vaccines in which the E protein is the main target of ZIKV neutralizing antibodies (Reichmuth et al., 2016; Zhao et al., 2016). This study highlighted that among the structural proteins, E protein is the most mutated, harboring 44 different mutations, followed by preM and capsid protein carrying 17 substitutions at different positions. The DIII of the E protein has been reported to be the main target of neutralizing antibodies against ZIKV (Zhao et al., 2016). Hence, mutations in the E protein are of great importance regarding their involvement in the host immune system and their neutralization. Not all mutations are in the favor of the viruses; hence, mutations sometimes harness the viruses to the antibodies and become easy targets for neutralizing antibodies. It was found that the coexisting mutations (I317 V and E393D) in the DIII domain of ZIKV E protein enhanced binding with the previously reported antibodies ZV64 and ZV67. Similarly, the mutations N139S/S139N play a vital role in the incidence of microcephaly. The 139N residue in the preM protein enhances the tropism of ZIKV for human progenitor cells and increases fetal microcephaly in mice (Yuan et al., 2017; Shah et al., 2022). On the other hand, ZIKV strains circulating in Asian countries carry the 139S residue in the preM protein, which is occasionally reported to be involved in ZIKV-related microcephaly (Wongsurawat et al., 2018). Based on previous reports (Pettersson et al., 2016) and the current study, it was observed that the majority of the isolates of American origin carry the 139N residue in their preM protein; hence, the ratio of ZIKV-related microcephaly is higher (2-12 per 10,000 live births). In contrast, all the isolates of European/Asian origin carry the 139S residue instead of the 139N residue in their preM protein; therefore, the incidence of ZIKV-related microcephaly in these regions is relatively lower (1 in 10,000 live births) (News-Medical-Life-Sciences, 2022).

Among the non-structural proteins, one of the biologically important mutations, 892 V/A, in NS1 has been reported to be involved in the secretions of ZIKV to the circulatory system, enhancing uptake of ZIKV by mosquitoes and participating in the phosphorylation of TBK1 and the transmission of the virus, thereby facilitating viral replication in humans (Xia et al., 2018). It was found that 892V/A is one of the re-occurring mutations in this study. Another important mutation was T233A (polyprotein T1026A) in the NS1, which has already been reported to destabilize the NS1 dimer and affect viral replication and pathogenesis. One of the re-occurring mutations in this study was M2634V, which was previously identified as neutral having no effect on the function of NS5 mutation. Similarly, mutations such as I3046A/T, K3107G/R, and N3167R/S in NS5 have been reported to inhibit intracellular interferon pathways and increase viral replication (Wang et al., 2016). The present study provided Insilco insights into the effect of different mutations on the structural stability and binding affinity of the ZIKV with the human-neutralizing antibodies. However, the limitation of our study is to provide the *in vitro* and *in vivo* experiments and human trials to further confirm the results of our study.

## 5. Conclusion

Being an RNA virus, the ZIKV continues to mutate rapidly and accumulate multiple mutations within its genome. In the current study, we reported rapid accumulations of various substitutions of amino acids in both structural and non-structural proteins of ZIKV circulating in different geographical regions of the world. Among the isolates of the ZIKV that were studied, one particular isolate (MN025403.1) from Guinea had the most mutations. This isolate had 111 substitutions and 6 deletions. The molecular docking and simulation approaches in this study verified that the mutated E protein enhanced the binding affinity of the ZIKV to the human-neutralizing antibodies. The study also highlighted the importance of monitoring mutations in the targeted genes of the ZIKV in order to improve the efficacy of vaccines and therapeutic applications. By understanding the impact of mutations on the structure and function of the virus, researchers can develop more effective antiviral drugs and vaccines.

## Data availability statement

The original contributions presented in this study are included in the article/Supplementary material, further inquiries can be directed to the corresponding author.

## Author contributions

ZX presented the concept, edited the manuscript, and supervised the project. AA and AS presented the concept, analyzed the data, and wrote the manuscript. AU conducted the mutational analysis. MS and SK performed the docking and simulation. FR, Al, and SL edited the manuscript and prepared the final draft. LX designed the figures. All authors contributed to the article and approved the submitted version.

## Footnotes

1
https://www.ncbi.nlm.nih.gov/

2
https://folding.biofold.org/i-mutant/i-mutant2.0.html

3
https://mupro.proteomics.ics.uci.edu

4
http://hdock.phys.hust.edu.cn/
