# Supplementary material for: Comparative mutational analysis of the Zika virus genome from different geographical locations and its effect on the efficacy of Zika virus-specific neutralizing antibodies
Source: Front Microbiol. 2023 Feb 22;14:1098323. doi: 10.3389/fmicb.2023.1098323 (PMC9992208; doi:10.3389/fmicb.2023.1098323)
Supplement: Supplementary file 1 [file Table_1.DOCX]

**Supplementary Table S1.** Mutational analysis of ZIKV isolates from different countries and territories

| **S.No** | **Country** | **Accession #** | **C (1-122)** | **preM (123-290)** | **E (291-794)** | **NSI (795-1146** | **NS2A (1147-1372)** | **NS2B( 1373-1502)** | **NS3 (1503-2119)** | **NS4A (2120-2245)** | **2K (2246-2269)** | **NS4B (2270-2520)** | **NS5 (2521-3423)** |
| --- | --- | --- | --- | --- | --- | --- | --- | --- | --- | --- | --- | --- | --- |
| 1 | Brazil | MH882543.1 |  | K242R, L275F, | K408R, | K985R |  | I1484V |  |  |  |  |  |
| 2 |  | MH882545.1 |  | K242R, L275F, | K408R, | K985R |  | 11484V |  |  |  |  |  |
| 3 |  | MH882546.1 |  | K242R, L275F, | K408R, | K985R |  | 11484V |  |  |  |  |  |
| 4 |  | MH882547.1 |  | K242R, L275F, | K408R, | K985R |  | 11484V |  |  |  |  |  |
| 5 |  | MH882548.1 |  | K242R, L275F, | K408R, | K985R |  | 11484V |  |  |  |  |  |
| 6 |  | KU321639.1 |  |  | V313I |  |  |  |  |  | I2295M | I2445M |  |
| 7 |  | KX197192.1 |  |  | V313I |  |  |  |  |  | I2295M | I2445M |  |
| 8 |  | MF073359.1 |  |  | K456R |  |  |  | G1623E |  |  |  |  |
| 9 |  | MK566202.1 |  |  | K456R |  | A1263V |  |  |  |  |  |  |
| 10 |  | KU497755.1 |  |  | S550T |  | L1259F |  |  |  |  |  |  |
| 11 |  | KU926310.2 |  |  | T625A |  |  |  |  | A2122T |  |  | V2688 |
| 12 |  | MN101548.1 |  |  | F672L |  |  |  | D1622G | F2123L,L2167M |  |  | Y2594H,S3162P |
| 13 |  | KY272991.1 |  |  |  | N889S | G1277S |  | K2039R |  |  |  |  |
| 14 |  | KX197192.1 |  |  |  | R893G,  Y916H |  |  | H1857Y |  |  |  |  |
| 15 |  | KU527068.1 |  |  |  | T1026A |  |  |  |  |  | T2509I |  |
| 16 |  | NK035889.1 |  |  |  | T1026A |  |  |  |  |  | T2509I |  |
| 17 |  | KX197205.1 |  |  |  | K985R |  |  | K985R |  |  |  | A2611T |
| 18 |  | MF073358.1 |  |  |  |  | T1176I,  I1180T,  M1327V |  | H1594Y |  |  |  |  |
| 19 |  | MF882527.1 |  |  |  |  | K1202R,  L1298V | A1428V | V1862I |  |  |  |  |
| 20 |  | MF882528.1 |  |  |  |  | K1202R,  L1298V | A1428V | V1862I |  |  |  |  |
| 21 |  | MF882529.1 |  |  |  |  | K1202R,  L1298V | A1428V | V1862I |  |  |  |  |
| 22 |  | MF882530.1 |  |  |  |  | K1202R,  L1298V | A1428V | V1862I |  |  |  | R2562H |
| 23 |  | MF882531.1 |  |  |  |  | K1202R,  L1298V | A1428V | V1862I |  |  |  | R2562H |
| 24 |  | MF882532.1 |  |  |  |  | K1202R,  L1298V | A1428V | V1862I |  |  |  | R2562H |
| 25 |  | MF882533.1 |  |  |  |  | K1202R,  L1298V | A1428V | V1862I |  |  |  | R2562H |
| 26 |  | MF882533.1 |  |  |  |  | K1202R,  L1298V | A1428V | V1862I |  |  |  | R2562H |
| 30 |  | MF882534.1 |  |  |  |  | K1202R,  L1298V | A1428V | V1862I |  |  |  | R2562H |
| 31 |  | MF882535.1 |  |  |  |  | K1202R,  L1298V | A1428V | V1862 |  |  |  | R2562H |
| 32 |  | MF882536.1 |  |  |  |  | K1202R,  L1298V | A1428V | V1862 |  |  |  |  |
| 33 |  | MF882538.1 |  |  |  |  | K1202R,  L1298V | A1428V | V1862 |  |  |  | R2562H |
| 34 |  | MF882539.1 |  |  |  |  | K1202R,  L1298V | A1428V | V1862 |  |  |  | R2562H |
| 35 |  | MF882540.1 |  |  |  |  | K1202R,  L1298V | A1428V | V1862 |  |  |  | R2562H |
| 36 |  | MF882541.1 |  |  |  |  | K1202R,  L1298V | A1428V | V1862 |  |  |  |  |
| 37 |  | MF882542.1 |  |  |  |  | K1202R,  L1298V | A1428V | V1862 |  |  |  | E2693G |
| 38 |  | MH513598.1 |  |  |  |  |  | I1398V | T2068M |  |  | S2455L |  |
| 39 |  | KU926309.1 |  |  |  |  |  | M1404I | K2039R |  |  |  |  |
| 40 |  | MH513600.1 |  |  |  |  |  |  |  |  |  | T2294A |  |
| 41 |  | MF073357.1 |  |  |  |  |  |  |  |  |  | I2295T |  |
| 42 |  | MT483911.1 |  |  |  |  |  |  |  |  |  | I2445M |  |
| 43 | Cambodia  COMBODIA | AMR39834.1 | A106T | A123V,S130N,N139S,L151M.L257F | M763V | E940K.V982A | L1274P | T1477A |  |  |  |  | V3392M,  M3403V |
| 44 |  | AOA14215B9.1 |  | A123V,S130N,N139S,L151M.L257F | M763V | E940K,  .V982A | L1274P | T1477A |  |  |  |  | V3392M,  M3403V |
| 45 |  | AWH65848.1 |  | A123V,S130N,N139S,L151M.L257F | M763V | E940K,  .V982A | L1274P | T1477A |  |  |  |  | V3392M,  M3403V |
| 46 |  | KU955593.1 |  | A123V,S130N,N139S,L151M.L257F | M763V | E940K,  .V982A | L1274P | T1477A |  |  |  |  | V3392M,  M3403V |
| 47 |  | MH158236.1 |  | A123V,S130N,N139S,L151M.L257F | M763V | E940K,  .V982A | L1274P | T1477A |  |  |  |  | V3392M,  M3403V |
| 48 |  | MZ008356.1 |  | L257F |  | E940K | 1204T |  |  |  |  |  | V2787A |
| 49 | Haiti | KU509998.3 |  | L257F |  | Y916H,  E940K |  |  | H1857Y |  |  | I2295M,  I2445M | M2634V, |
| 50 |  | MF384325.1 |  | L257F |  | E940K,  M1143V, |  |  |  |  |  |  | M2634V,  I2842V,  D3398E |
| 51 |  | MF783072.1 |  | L257F |  | E940K,  M1143V, |  |  |  |  |  |  | M2634V,  I2842V,  D3398E |
| 52 |  | MF783073.1 |  | L257F | V313I | Y916H,  E940K, |  |  | H1857Y |  |  | I2295M,  I2445M | M2634V, |
| 53 |  | MN566104.1 |  | L257F |  | E940K,  M1143V |  |  |  |  |  |  | M2634V, D3398E |
| 54 |  | MN566105.1 |  | L257F |  | E940K,  M1143V |  |  |  |  |  |  | M2634V, I2842V, D3398E |
| 55 |  | MN566106.1 |  | L257F |  | E940K,  M1143V |  |  |  |  |  |  | M2634V, I2842V, D3398E |
| 56 |  | MN566107.1 |  | L257F |  | E940K,  M1143V |  |  |  |  |  |  | M2634V, I2842V, D3398E |
| 57 |  | MN566108.1 |  | L257F |  | E940K,  M1143V |  |  |  |  |  |  | M2634V, I2842V, D3398E |
| 58 |  | MN577543.1 |  | L257F |  | E940K,**R1118W** |  | **D1434E** | D1826N,S1858F |  |  |  | M2634V**,D3193N,R3195T,F3196V** |
| 59 |  | MN577544.1 |  | L257F | G472R, | **W862M,**  E940K, E1052R,  **R1118W,**  **L1164I** | I1179N,T1305K |  | L1738P,A1922V,  K1968N |  |  |  | M2634V,Q3253H,D3254N,E3255G,A3266S,T3353A,L3413F,G3414C,E3416Y,G3417F |
| 60 | Ecuador | AOR82892.1 |  | L257F |  | E940K,  M1143V |  |  |  |  |  |  | M2634V |
| 61 |  | AOR82893.1 |  | L257F |  | E940K,  M1143V |  |  |  |  |  |  | M2634V |
| 62 |  | KX879603.1 |  | L257F |  | E940K,  M1143V |  |  |  |  |  |  | M2634V |
| 63 |  | KX879604.1 |  | L257F |  | Y916H,  E940K,  M1143V |  |  |  |  |  |  | M2634V |
| 64 | French Poly | AOA024B7W1.1 |  | L257F |  | E940K, |  |  |  |  |  |  |  |
| 65 |  | KJ776791.2 |  | L257F |  | E940K, |  |  |  |  |  |  |  |
| 66 |  | KX369547.1 |  |  |  |  |  |  |  |  |  |  |  |
| 67 |  | KY766069.1 |  | L257F |  | E940K, |  |  |  |  |  |  |  |
| 68 | Guinea | MN025403.1 | K6R,  S25N,  F27L,  K101R,  V110I | V125I,  N139S,E143K,  P148A  M153V,Y157H,I158V,R246K,L251F,A260V, ,A262V,L257F,A260V,A262V | A410T.I459V,S575F,I607V, E683D,A727V,L728F,M763V,M777T,L785M, | D846E,R863K,S886P, E940K,V956I,V988A,K1007R,I1030V,M1058V | I1180M,A1204V,I1226V,D1270E,V1289A,T1297A,L1354M | D1461E,T1477A | S1558A,H1594L,I1658V,R1671V,K1687R,T1717K,V1722A,T1753I,K1860R,V1862I,H1902N,V1909I,L1974M,R2085K,H2086Y, | F2123LE2127D | L2282I,R2289K,A2293T,I2453V,S2455L, | F2318L | Y2594H,I2598V,K2621R,A2679T,L2715M,Y2722H,T2749I,V2787A,N2800R,S2807N,I2842V,S2896N,H2909R,E2935V,Q2969H,V3039I,S3044N,I3046A,R3065K,K3080E,I3089V,K3107G,Q3154H,R3161K,S3162P,N3167R,N3172D,D3223S,S3304A,V3333M,T3353K,N3387D |
| 69 | Guatemala | KU501216 |  | L257F | V346I, | G894A,  E940K |  |  | M2074L |  |  |  | M2634V,K2694P,R3045C |
| 70 |  | KU501217.1 |  | L257F | V346I | G894A, E940K |  |  | M2074L |  |  |  | M2634V,K2694P,R3045C |
| 71 | Cuba | MF438286.1 | A120V | E143K  R211W, L257F |  | V840I, E940N,M1143V | V1188I, | T1477A | T2069I | A2357T |  | A2470T | M2634V,  I2842V,D3398E |
| 72 | Honduras | KX694534.1 |  | L257F | K733R | G894A,  E940K,  Y969S | G1370R |  | M2074L |  |  |  | M2634V,  R3045C |
| 73 |  | KY328289.1 |  | L257F |  | G894A,  E940K |  |  | M2074L |  |  |  | M2634V,  R3045C |
| 74 | Italy | KU853012.1 |  |  | A791V | E940K,M1143V |  |  |  |  |  |  | M2634V,I2842V,D3398E |
| 75 |  | KU853013.1 |  |  |  | E940K, M1143V |  |  |  |  |  |  | M2634V, I2842V, D3398E |
| 76 |  | KX269878.1 |  |  |  | E940K, M1143V |  |  |  |  |  |  | M2634V, I2842V, D3398E |
| 77 |  | KY003153.1 |  |  |  | E940K, |  |  |  | F2123L |  |  | Y2594H, M2634V, I2842V |
| 78 | Martinique | KU647676.1 | D107E | L257F |  | E940K,  R1118W | I1226T |  |  |  |  |  | M2634V,T3353A |
| 79 | Micronesia | EU545988.1 | K6E.K7E,S8I,G9R,G10R,F11I,A106T, | A123V,N139S,L257F, | T330A,M763V,M777T | E820D, E940K,  ,V982A | A1302T |  | L1614P,Q1898L,H1902N,R2062K,H2086Y, | K2161E, | V2259I | I2367M,P2433H,Q2434R | V3392M |
| 80 | Panama | MN100039.1 | D107E.E209K | ,L257F |  | S852F,  R897K,  E940K,  R1118W |  |  |  |  |  |  | M2634V, T3353A |
| 81 |  | MN124090.1 | D107E, | ,L257F |  | S852F,  E940K,  R1118W,A1263V |  |  |  |  |  |  | M2634V,  T3353A |
| 82 |  | MN124091.1 | D107E, E209K | ,L257F | M358I,A712V | S852F.  R897K,  E940K,  R1118W |  |  |  |  |  |  | M2634V, T3353A |
| 83 | Nigeria | KU963574.2 | S25N.F27L,K101R,T108A,V110I, G114S,A120V, | V125I,  N139S,E143K,P148V,M153V,Y157H,I158V, ,R246K,L257F,A260V,A262V, | (446-451,TGHETD),I459V,A517S,G522E,I607V,T625R, E683D,T696I,A727V,L728F,N763V,M777T,L785M, | V815I,K827R,D846E,R863K,Q896R,E940K,V988A,1030V,M1058V, | I1180M,V1186A,I1191V,A1204V,I1226V,D1270E,I1275V,V1289A,T1297A,I1329I,L1354I, | S1417I,D1461E,T1477A, | S1558A,H1594L,R1609K,R1671K,K1687R,T1717K,V1862I,H1902N,V1909I,L1974M,R2085K,H2086Y, | F22123L,E2127D, |  | L2282I,S2083A.R2289K,A2293T,I2295V,F2318L,I2367M,V2449A,I2453V,S2455L, , | Y2594H,I2698V,K2621R,A2679T,L2715M,T2749I,V2787A,N2800R,S2807N,I2842V,Q2893R,S2896N,H2909R,A2969H,V3039I,S3044N,I3046A,R3050K,K3080E,I3089V,K3107G,Q3154H,R3116K,S3162P,N3067R,D3223S,S3304A,V3307A,T3328N,V3333M,E3348G,N3387D,K3401R |
| 84 | Singapore | KY241765.1 | N119S | L257F |  | E940K,  V988A |  |  | H2086Y, |  | , |  |  |
| 85 |  | KY241787 | N119S | L257F |  | E940K, V988A |  |  | H2086Y, |  |  |  |  |
| 86 | South Korea | APG56457.1 |  | L257F |  | E940K,  M1143V |  |  |  |  |  |  | M2634V,  I3046T, E3149G |
| 87 |  | APG56458.1 |  | A123V.N139S, L257F | M763V, M777T | V840I,  E940K, | I1205T, | V1439I, | H1902N, T2068M, H2086Y | I2157M,  I2367M, |  |  | I2598V,H2909N,S3162P,V3392M |
| 88 |  | KY553111.1 |  | A123V, N139S, L257F | V443G,M763V,M777T | V840I,  E940K, | I1205T, | V1439I, | R1609M, H1902N,T2068M,H2086Y | I2157M, I2367M |  |  | I2598V, H2909N,S3162P,V3392M |
| 89 | Senegal | AMR39832.1 | S25N,F27L,K101R,V110I | V125I,N139S,E143K,P148A,M153V,Y157H,I158V,R246K,L257F,A260V,A262V, | A410T,I459V,S575F,I610V,E683D,A727V,A728F,M763V,M777T,L785M, | D846E,R863K,E940K,V956I,V988A,K1007R,I1030V,M1058V, | 1180M,I1191V,A1204V,I1226V,D1270E,I1275V,V1289A,T1297A,L1354I, | D1461E,T1477A, | S1558A,H1594L,R1671K,T1676A,K1687R,T1717K,V1862I,H1902N,V1909I,I1962V,L1974M,R2085K,H2086Y, | F2123L,E2127D, |  | L2282I,S2283A,R2289K,A2293T,I2295M,F2308L,I2367M,I2453V,S2455L,  , | Y2594H,I2598V,K2621R,A2679T,L2705M,Y2722H,T2749I,D2765E,V2727A,N2800R,S2807N,I2842V,S2896N,H2909R,Q2969H,V3039I,S3044N.  I3046A,R3050K,R3065K,K3080E,I3089V,K3107G,Q3054H,R3161K,S3162P,N3167R,D3223S,S3304A,**V3307I,**V3333M,N3387D,3401F |
| 90 |  | AMR39833.1 |  | V125I,N139S,E143K,P148A,M153V,Y157H,I158V,R246K,L257F,A260V,A262V | A410T,I459V,S575F,I610V,E683D,A727V,A728F,M763V,M777T,L785M, | D846E,R863K,E940K,V956I,V988A,K1007R,I1030V,M1058V, | 1180M,I1191V,A1204V,I1226V,D1270E,I1275V,V1289A,T1297A,L1354I, | D1461E,T1477A, | S1558A,H1594L,R1671K,T1676A,K1687R,T1717K,V1862I,H1902N,V1909I,I1962V,L1974M,R2085K,H2086Y, | F2123L,E2127D, |  | L2282I,S2283A,R2289K,A2293T,I2295M,F2308L,I2367M,I2453V,S2455L, | Y2594H,I2598V,K2621R,A2679T,L2705M,Y2722H,T2749I,D2765E,V2727A,N2800R,S2807N,I2842V,S2896N,H2909R,Q2969H,V3039I,S3044N,  I3046A,R3050K,R3065K,K3080E,I3089V,K3107G,Q3054H,R3161K,S3162P,N3167R,D3223S,S3304A,**V3307I,**V3333M,N3387D,3401F |
| 91 |  | AMR39836.1 | S25N,F27L,K101R,V110I | V125I,N139S,E143K,P148A,M153V,Y157H,I158V,R246K,L257F,A260V,A262V | A410T,I459V,S575F,I610V,E683D,A727V,A728F,M763V,M777T,L785M, | D846E,R863K,E940K,V956I,V988A,K1007R,I1030V,M1058V, | 1180M,I1191V,A1204V,I1226V,D1270E,I1275V,V1289A,T1297A,L1354I, | D1461E,T1477A, | S1558A,H1594L,R1671K,T1676A,K1687R,T1717K,V1862I,H1902N,V1909I,I1962V,L1974M,R2085K,H2086Y, | F2123L,E2127D, |  | L2282I,S2283A,R2289K,A2293T,I2295M,F2308L,I2367M,I2453V,S2455L, | Y2594H,I2598V,K2621R,A2679T,L2705M,Y2722H,T2749I,D2765E,V2727A,N2800R,S2807N,I2842V,S2896N,H2909R,Q2969H,V3039I,S3044N, I3046A,R3050K,R3065K,K3080E,I3089V,K3107G,Q3054H,R3161K,S3162P,N3167R,D3223S,S3304A,**V3307I,**V3333M,N3387D,3401F |
| 92 |  | KU955591.1 | S25N,F27L,K101R,V110I | V125I,N139S,E143K,P148A,M153V,Y157H,I158V,R246K,L257F,A260V,A262V | A410T,I459V,S575F,I610V,E683D,A727V,A728F,M763V,M777T,L785M, | D846E,R863K,E940K,V956I,V988A,K1007R,I1030V,M1058V, | 1180M,I1191V,A1204V,I1226V,D1270E,I1275V,V1289A,T1297A,L1354I, | D1461E,T1477A, | S1558A,H1594L,R1671K,T1676A,K1687R,T1717K,V1862I,H1902N,V1909I,I1962V,L1974M,R2085K,H2086Y, | F2123L,E2127D, |  | L2282I,S2283A,R2289K,A2293T,I2295M,F2308L,I2367M,I2453V,S2455L , | Y2594H,I2598V,K2621R,A2679T,L2705M,Y2722H,T2749I,D2765E,V2727A,N2800R,S2807N,I2842V,S2896N,H2909R,Q2969H,V3039I,S3044N, I3046A,R3050K,R3065K,K3080E,I3089V,K3107G,Q3054H,R3161K,S3162P,N3167R,D3223S,S3304A,**V3307I,**V3333M,N3387D,3401F |
| 93 |  | KU955592.1 | S25N,F27L,K101R,V110I | V125I,N139S,E143K,P148A,M153V,Y157H,I158V,R246K,L257F,A260V,A262V | A410T,I459V,S575F,I610V,E683D,A727V,A728F,M763V,M777T,L785M, | D846E,R863K,E940K,V956I,V988A,K1007R,I1030V,M1058V, | 1180M,I1191V,A1204V,I1226V,D1270E,I1275V,V1289A,T1297A,L1354I, | D1461E,T1477A, | S1558A,H1594L,R1671K,T1676A,K1687R,T1717K,V1862I,H1902N,V1909I,I1962V,L1974M,R2085K,H2086Y, | F2123L,E2127D, |  | L2282I,S2283A,R2289K,A2293T,I2295M,F2308L,I2367M,I2453V,S2455L, | Y2594H,I2598V,K2621R,A2679T,L2705M,Y2722H,T2749I,D2765E,V2727A,N2800R,S2807N,I2842V,S2896N,H2909R,Q2969H,V3039I,S3044N, I3046A,R3050K,R3065K,K3080E,I3089V,K3107G,Q3054H,R3161K,S3162P,N3167R,D3223S,S3304A,**V3307I,**V3333M,N3387D,3401F |
| 94 |  | KU955595.1 | S25N,F27L,K101R,V110I | V125I,N139S,E143K,P148A,M153V,Y157H,I158V,R246K,L257F,A260V,A262V | A410T,I459V,S575F,I610V,E683D,A727V,A728F,M763V,M777T,L785M, | D846E,R863K,E940K,V956I,V988A,K1007R,I1030V,M1058V, | 1180M,I1191V,A1204V,I1226V,D1270E,I1275V,V1289A,T1297A,L1354I, | D1461E,T1477A, | S1558A,H1594L,R1671K,T1676A,K1687R,T1717K,V1862I,H1902N,V1909I,I1962V,L1974M,R2085K,H2086Y, | F2123L,E2127D, |  | L2282I,S2283A,R2289K,A2293T,I2295M,F2308L,I2367M,I2453V, S2455L, | I3046A,R3050K,R3065K,K3080E,I3089V,K3107G,Q3054H,R3161K,S3162P,N3167R,D3223S,S3304A,**V3307I,**V3333M,N3387D,3401F |
| 95 |  | MF510857.1 | S25N,F27L,K101R,V110I | V125I,N139S,E143K,P148A,M153V,Y157H,I158V,R246K,L257F,A260V,A262V | A410T,I459V,S575F,I610V,E683D,A727V,A728F,M763V,M777T,L785M, | D846E,R863K,E940K,V956I,V988A,K1007R,I1030V,M1058V, | 1180M,I1191V,A1204V,I1226V,D1270E,I1275V,V1289A,T1297A,L1354I, | D1461E,T1477A, | S1558A,H1594L,R1671K,T1676A,K1687R,T1717K,V1862I,H1902N,V1909I,I1962V,L1974M,R2085K,H2086Y, | F2123L,E2127D, |  | L2282I,S2283A,R2289K,A2293T,I2295M,F2308L,I2367M,I2453V,S2455L, S2455L | ,Y2594H,I2598V,K2621R,A2679T,L2705M,Y2722H,T2749I,D2765E,V2727A,N2800R,S2807N,I2842V,S2896N,H2909R,Q2969H,V3039I,S3044N,  I3046A,R3050K,R3065K,K3080E,I3089V,K3107G,Q3054H,R3161K,S3162P,N3167R,D3223S,S3304A, **,**V3333M,N3387D, |
| 96 | Malaysia | ANK57896.1 |  | A123V,N139S,E143K,M153V,L257V | T446I,E683D,M763V,M777T, , | E940K,V982A,M1058V, | A1263V,V1289A, |  | H1902N,L1974M,H2086Y, |  | S2283G, | I2295M,  F2318L,I2367M,V2449I,V2453I,S2455L, | M2634T,P2659S,T2749I,V2787A,M2795L,I2802V,S2896N,I3046T,R3050K,K31O7R,S3162P,N3167S,D3223S,H3233Y,H3239Y,N3387D. |
| 97 |  | KX377336.1 |  | A123V,N139S,E143K,M153V,L257V | T446I,E683D,M763V,M777T, | E940K,V982A,M1058V, | A1263V,V1289A, |  | H1902N,L1974M,H2086Y, |  | S2283G, | I2295M,  F2318L,I2367M,V2449I,V2453I,S2455L, |  |
| 98 | Suriname | KY348640.1 |  | L256F |  | V887A,E940K | I1180V, |  | M2038I, |  |  |  | M2634V,M3403V |
| 99 | Nicaragua | MN577550.1 | T08A |  | L257F,  T450A,T603A, , | G894A,E940K, |  |  | M2074L, |  |  |  | M2634V,R3045C |
| 100 | Mexico | APC60215.1 |  | L257F |  | G894A. E940K |  |  | V1542I.M2074L |  |  |  | M2634LR3045C,P3047S |
| 101 |  | APC60216.2 |  | L257F |  | G894A, E940K |  |  | V1542I M2074L |  |  |  | M2634LR3045C P3047S |
| 102 |  | KU922923.1 | D107E | L257F.P287C |  | E940K. R1118W | I1226T |  | A1608E |  |  |  | M2634LH2809QG3030VT3353A |
| 103 |  | KU922960.1 | D107E | L257F |  | E940K, R1118W | I1226T |  | A1608E |  |  |  | M2634LH2809Q G3030V  T3353A |
| 104 |  | KX856011.1 |  | L257F |  | G894A, E940K |  |  | V1542I, M2074L |  |  |  | M2634LR3045CS3162P |
| 105 |  | KY120348.1 |  | L257F |  | G894A, E940K |  |  | V1542I, M2074L |  |  |  | M2634LR3045CP3047S |
| 106 |  | KY120349.2 |  | L257F |  | G894A, E940K |  |  | V1542I, M2074L |  |  |  | M2634LR3045CP3047S |
| 107 |  | KY631493.1 |  | L257F |  | I872V, G894A, E940K |  |  | V1542I, M2074L |  |  |  | M2634LR3045C |
| 108 |  | KY631494.1 |  | L257F |  | I872V, G894A,  E940K |  |  | V1542I, M2074L |  |  |  | M2634LR3045C |
| 109 |  | MH900227.1 |  | L257F | H691Y | G894A, E940K | A1263V |  | V1542I,  T1879I, M2074L |  |  |  | M2634LR3045CP3047S |
| 110 |  | MT507047.1 |  | M177T, L257F | N254K | G894A, K1059T, T1096A |  |  | V1542I, M2074L |  |  |  | M2634LR3045CP3047S |
| 111 |  | MT507048.1 |  | M177T, L257F |  | G894A, E940K |  |  | V1542I, M2074L |  |  |  | M2634LR3045CP3047S |
| 112 |  | MT507049.1 | I50T, | M177T, L257F | N254K | G894A | A1263V |  | V1542I, M2074L |  |  |  | M2634LR3045CP3047S |
| 113 |  | MT507050.1 | I50T | M177T, L257F | N254K | G894A | A1263V |  | V1542I, M2074L |  |  |  | M2634LR3045  P3047S |
