# Supplementary material for: Comparative mutational analysis of the Zika virus genome from different geographical locations and its effect on the efficacy of Zika virus-specific neutralizing antibodies
Source: Front Microbiol. 2023 Feb 22;14:1098323. doi: 10.3389/fmicb.2023.1098323 (PMC9992208; doi:10.3389/fmicb.2023.1098323)
Supplement: Supplementary file 2 [file Table_2.DOCX]

**Supplementary Table S2.** Mutational effects on structure stability of structural and nonstructural proteins of zika virus isolates

| Protein | Top reoccurring mutation | MUpro  Predicted Outcome  ∆∆G | I-mutant  Predicted Outcome  ∆∆G |
| --- | --- | --- | --- |
| C | A106T | -1.5637 Decrease Stability | -0.46 Decrease Stability |
|  | D107E | -1.2049 Decrease Stability | -0.97 Decrease Stability |
| preM | A123V | 0.1203 Increase Stability | -0.64 Decrease Stability |
|  | S130N | -0.8003 Decrease Stability | -1.87 Decrease Stability |
|  | N139S | -2.0300 Decrease Stability | -0.13 Decrease Stability |
|  | L151M | -0.4748 Decrease Stability | 0.24 Decrease Stability |
|  | K242R | -1.0375 Decrease Stability | -0.15 Decrease Stability |
|  | L257F | -0.9825 Decrease Stability | -0.24 Decrease Stability |
| E | V313I, | -0.5185 Decrease Stability | -1.25 Decrease Stability |
|  | K408R, | -0.4530 Decrease Stability | -0.59 Decrease Stability |
|  | M763V, | -0.8825 Decrease Stability | 0.18 Decrease Stability |
|  | M777T | -1.6735 Decrease Stability | -0.77 Decrease Stability |
| NS1 | Y916H ( 122) | -1.4005 Decrease Stability | -0.19 Decrease Stability |
|  | E940L (146) | -0.4144 Decrease Stability | 0.**09 Increase stability** |
|  | V982A (188) | -1.3745 Decrease Stability | -0.34 Decrease Stability |
|  | K985R ( 191) | -0.1772 Decrease Stability | -1.10 Decrease Stability |
|  | R1118W( 324) | -1.6628 Decrease Stability | -0.63 Decrease Stability |
|  | M1143V ( 349) -0.9469 Decrease Stability -0.27 Decrease Stability | | |
| NS2A | K1202R (56) | -0.4407 Decrease Stability | -1.20 Decrease Stability |
|  | L1274P ( 128) | -2.3583 Decrease Stability | -1.77 Decrease Stability |
|  | L1289V ( 152) | -1.0260 Decrease Stability | -0.96 Decrease Stability |
| NS2B | A1428V ( 56) | -0.9712 Decrease Stability | 0.67 Decrease Stability |
|  | T1477A ( 105) | -1.1504 Decrease Stability | -3.81 Decrease Stability |
|  | I1484V ( 112) | -0.3605 Decrease Stability | -0.58 Decrease Stability |
| NS3 | H1857Y (355) | 0.2125 Increase stability | -0.13 **Increase stability** |
|  | V1862I (360) | -0.3331 Decrease Stability | -1.54 Decrease Stability |
|  | H1902N ( 400) | -0.1659 Decrease Stability | -0.29 **Increase stability** |
|  | M2074L ( 572) | -0.8857 Decrease Stability | -0.11 Decrease Stability |
|  | H2086Y (584) | -0.1827 Decrease Stability | 0.15 **Increase stability** |
| NS4A | F2123L (4) | -0.7418 Decrease Stability | -0.28 Decrease Stability |
| NS4B | I2295M,(26) | -0.7875 Decrease Stability | -1.26 Decrease Stability |
|  | I2367M, (98) | -1.2370 Decrease Stability | -2.36 Decrease Stability |
|  | I2445M (176) | -1.0758 Decrease Stability | -1.42 Decrease Stability |
| NS5 | R2562H (41) | -1.2749 Decrease Stability | -0.91 Decrease Stability |
|  | M2634V (114) | -0.3603 Decrease Stability | -0.07 **Increase Stability** |
|  | I2842V (322) | -1.2639 Decrease Stability | -0.62 Decrease Stability |
|  | R3045C (525) | -0.7683 Decrease Stability | -1.16 Decrease Stability |
|  | S3162P (642) | -1.0561 Decrease Stability | -0.21 **Increase stability** |
|  | T3353A (833) | -0.8908 Decrease Stability | -1.60 Decrease Stability |
|  | V3392M (872) | -1.2331 Decrease Stability | 0.25 Decrease Stability |
|  | D3398E (878) | -0.5791 Decrease Stability | -1.50 **Increase stability** |
|  | M3403V (883) | -0.9062 Decrease Stability | -0.93 Decrease Stability |

The top reoccurring substitutions in structural and nonstructural protein showed that expect the A123V and H1857Y substitutions in preM and NS3 protein respectively, all other substitutions decreased the structural stability of the corresponding proteins of the zika virus in the present study.
